# The need for tumor surveillance of children and adolescents with cancer predisposition syndromes: a retrospective cohort study in a tertiary-care children’s hospital

**DOI:** 10.1007/s00431-021-04347-x

**Published:** 2021-12-23

**Authors:** Simon Huber, Mareike Schimmel, Désirée Dunstheimer, Karolina Nemes, Markus Richter, Joachim Streble, Kurt Vollert, Ulrike Walden, Michael C. Frühwald, Michaela Kuhlen

**Affiliations:** 1 Paediatric and Adolescent Medicine, University Medical Center, Stenglinstr. 2, 86156 Augsburg, Germany; 2 Department of Diagnostic and Interventional Radiology and Neuroradiology, University Medical Center, Stenglinstr. 2, 86156 Augsburg, Germany; 3 Swabian Children’s Cancer Center, University Medical Center Augsburg, Stenglinstr. 2, 86156 Augsburg, Germany

**Keywords:** Cancer predisposition, Children, Surveillance, Children’s hospital, Recommendations

## Abstract

Expert recommendations for the management of tumor surveillance in children with a variety of cancer predisposition syndromes (CPS) are available. We aimed (1) at identifying and characterizing children who are affected by a CPS and (2) at comparing current practice and consensus recommendations of the American Association for Cancer Research workshop in 2016. We performed a database search in the hospital information system of the University Children’s Hospital for CPS in children, adolescents, and young adults and complemented this by review of electronic patients’ charts. Between January 1, 2017, and December 3, 2019, 272 patients with 41 different CPS entities were identified in 20 departments (144 [52.9%] male, 128 [47.1%] female, median age 9.1 years, range, 0.4–27.8). Three (1.1%) patients died of non-malignancy-associated complications of the CPS; 49 (18.0%) patients were diagnosed with malignancy and received regular follow-up. For 209 (95.0%) of the remaining 220 patients, surveillance recommendations were available: 30/220 (13.6%) patients received CPS consultations according to existing consensus recommendations, 22/220 (10.0%) institutional surveillance approaches were not complying with recommendations, 84/220 (38.2%) patients were seen for other reasons, and 84/220 (38.2%) were not routinely cared for. Adherence to recommendations differed extensively among CPS entities.

*Conclusion*: The spectrum of CPS patients at our tertiary-care children’s hospital is manifold. For most patients, awareness of cancer risk has to be enhanced and current practice needs to be adapted to consensus recommendations. Offering specialized CPS consultations and establishing education programs for patients, relatives, and physicians may increase adherence to recommendations.

**Table:** 

**What is Known:**
• *A wide spectrum of rare syndromes manifesting in childhood is associated with an increased cancer risk.*
• *For many of these syndromes, expert recommendations for management and tumor surveillance are available, although based on limited evidence.*
**What is New:**
• *Evaluating current practice, our data attest significant shortcomings in tumor surveillance of children and adolescents with CPS even in a tertiary-care children’s hospital.*
• *We clearly advocate a systematic and consistent integration of tumor surveillance into daily practice.*

## Introduction

Hereditary cancer predisposition is increasingly recognized these days [1–5]. More than 100 cancer predisposition syndromes (CPS) are currently known [6–8]. The spectrum is diverse, and for the time being, diseases are grouped provisionally as DNA repair and telomere biology disorders, immunodeficiencies, RASopathies, overgrowth syndromes, developmental disorders, chromosomal anomalies, and metabolic and endocrine disorders.

With rapidly increasing use and advances of genome sequencing, genome-wide chromosomal microarrays and long-read technologies, bioinformatic analyses, functional in vitro and in vivo assessment, and the availability of large databases, new genes and syndromes will be discovered and more children carrying cancer susceptibility variants will be identified [9]. Although a sequence variant may be clearly predisposing to cancer in general, the specific relevance for the respective carrier remains ambiguous in most cases [10]. This conflict goes beyond the scope of individuals already afflicted by cancer, but involves thus far unaffected children and adolescents as well.

By definition, individuals with CPS carry a statistically increased risk of developing cancer [11]. The identification of individuals carrying cancer predisposing variants is closely connected with the hope to substantially improve outcome by early detection of tumors and, potentially, cancer prevention [12]. Due to the rarity of most CPS in childhood, our knowledge of penetrance and expressivity is limited, [10] and evidence-based guidelines for referral, diagnosis, and management of carriers are missing for most [12]. Recommendations concerning surveillance should focus on the types of cancer(s) to which the individual is most at risk, and the time frame of greatest risk [1, 11]. The benefit of early detection needs to be weighed against the physical and psychological burden of repeated examinations placed on the patients and their families [1, 12, 13].

To this end, the American Association for Cancer Research (AACR) conducted a workshop in 2016, aiming at developing consensus recommendations for cancer surveillance in children and adolescents with CPS [11]. The group decided to recommend surveillance if the risk of developing cancer during the first 20 years of life exceeds 5% and effective screening modalities exist. Surveillance recommendations for conditions with a cancer risk between 1 and 5% were decided by the expert panel on an individual basis [11]. It should not go unmentioned that — in children — efficacy of many cancer surveillance protocols for rare CPS including screening modalities still needs to be confirmed. Currently, a survival benefit for children undergoing surveillance has only been demonstrated for Li-Fraumeni syndrome and constitutional mismatch repair deficiency [14, 15].

Surveillance protocols are primarily designed for asymptomatic carriers of variants with a predisposition to develop cancer. Yet, other serious conditions may be caused by the wide spectrum of rare diseases for which these children may already be cared for by various pediatric specialists.

We conducted a retrospective single-center cohort study in a tertiary-care children’s hospital to elucidate current practice of surveillance in children and adolescents carrying cancer predisposing variants. Aims of the study were (1) to determine the number and clinical characteristics of children and adolescents affected by CPS in a tertiary-care children’s hospital, and (2) to compare current hospital practice with the surveillance recommendations and, if necessary, adjust practice to recommendations.

## Materials and methods

### Identification of children, adolescents, and young adults diagnosed with CPS

We compiled a list of CPS manifesting in childhood and adolescence by using the AACR recommendations [11, 16–28] and reviews on CPS in children and adolescents [6–8]. An ICD-10 code was assigned to each CPS based on the information given in “orphanet” [29] (Table 1).

**Table 1:**
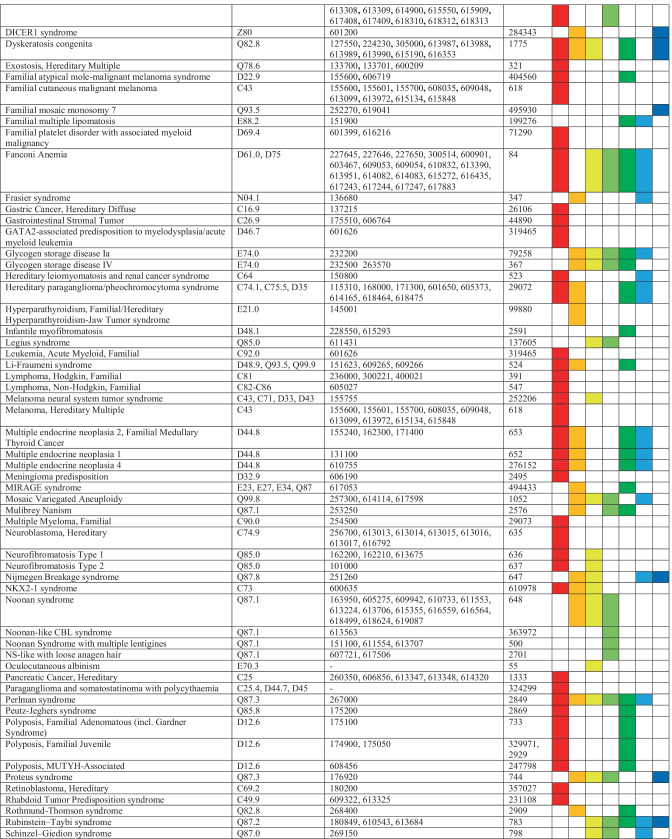

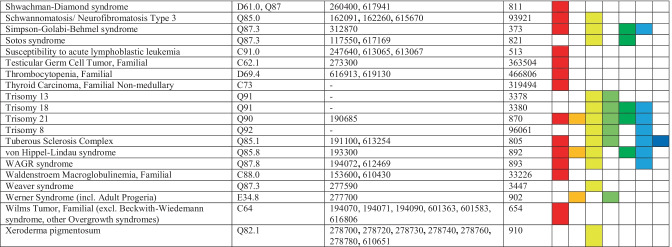
List of cancer predisposition syndromes (CPS) manifesting in childhood and adolescence compiled by using the AACR recommendations and reviews on CPS in children and adolescents and assigned ICD-10 codes, OMIM® numbers and ORPHA codes. The right columns indicate the pediatric specialties which were contacted for eligible patients

We searched the hospital information system (ORBIS® v. 08,043,302.11210.DACHL, Agfa Health Care N.V., Belgium) for patients coded with any of the ICD-10 codes as primary or secondary diagnosis across all departments of the University Medical Center (UMC), Augsburg. In case a specific ICD-10 code was listed in “orphanet,” the general code was used instead (e.g., D12 instead of D12.6) in order to maximize search results. Results were checked for plausibility comparing ICD-10 codes and text entries. Patients were removed if incorrectly coded based on the text entry.

Each CPS was assigned to at least one of seven pediatric departments (i.e., cardiology, endocrinology, gastroenterology, hemato-oncology, nephrology, neurology, pulmonology) at the University Children’s Hospital that care for affected patients due to concomitant symptoms and conditions (Table 1). We provided the relevant set of CPSs to the senior physicians of those specialties and asked to identify eligible patients.

We merged data to remove duplicate cases. Patients for whom the diagnosis of a CPS was not confirmed by manual review of electronic patients’ charts were excluded. Finally, we reviewed medical reports of each patient to assess demographic data, patient characteristics, tumor diagnosis if applicable, extent of tumor surveillance (including frequency of appointments during the study period, type of examinations performed, and schedule of follow-up), and date and cause of death if applicable.

This analysis included (i) children, adolescents, and young adults treated at the University Children’s Hospital and (ii) aged < 18 years treated at non-pediatric departments of the UMC Augsburg, who were seen between January 1, 2017, and December 31 2019.

The study received approval by the Internal Review Board of the University Hospital, Augsburg, Germany.

### Analyzing current surveillance strategies in patients with CPS

We assigned patients to the respective department where they presented most frequently to identify the (pediatric) specialist responsible for primary treatment. For clarity and unambiguousness, the departments of pediatric immunology, pediatric rheumatology, developmental pediatrics, pediatric radiology, as well as pediatric emergency care, pediatric surgery, general pediatrics, neonatology, and intensive care were combined into a category of “other pediatric departments,” and the non-pediatric departments of dermatology, ophthalmology, and ear-nose-throat medicine to “non-pediatric” departments.

According to the published literature, CPSs were classified as entities in which surveillance (i) is recommended, (ii) is recommended depending on the affected gene/variant underlying the CPS, and (iii) is not recommended. We compared the adherence to these surveillance recommendations by assigning each patient to one of four groups: (1) patients regularly cared for in a specialized CPS program by the department of pediatric hemato-oncology (herein referred to as consensus recommendations), (2) patients regularly presenting to non-oncological departments for CPS-specific surveillance and coexisting conditions (herein referred to as institutional surveillance), (3) patients regularly presenting to non-oncological departments for coexisting conditions but not receiving CPS-specific surveillance, and (4) patients not regularly presenting to the University Hospital Augsburg.

In a final step, guidelines from published surveillance recommendations were compared to current practice at the University Hospital Augsburg and classified as (i) adhering to consensus recommendations or (ii) not complying with consensus recommendations.

## Results

**Fig. 1:**
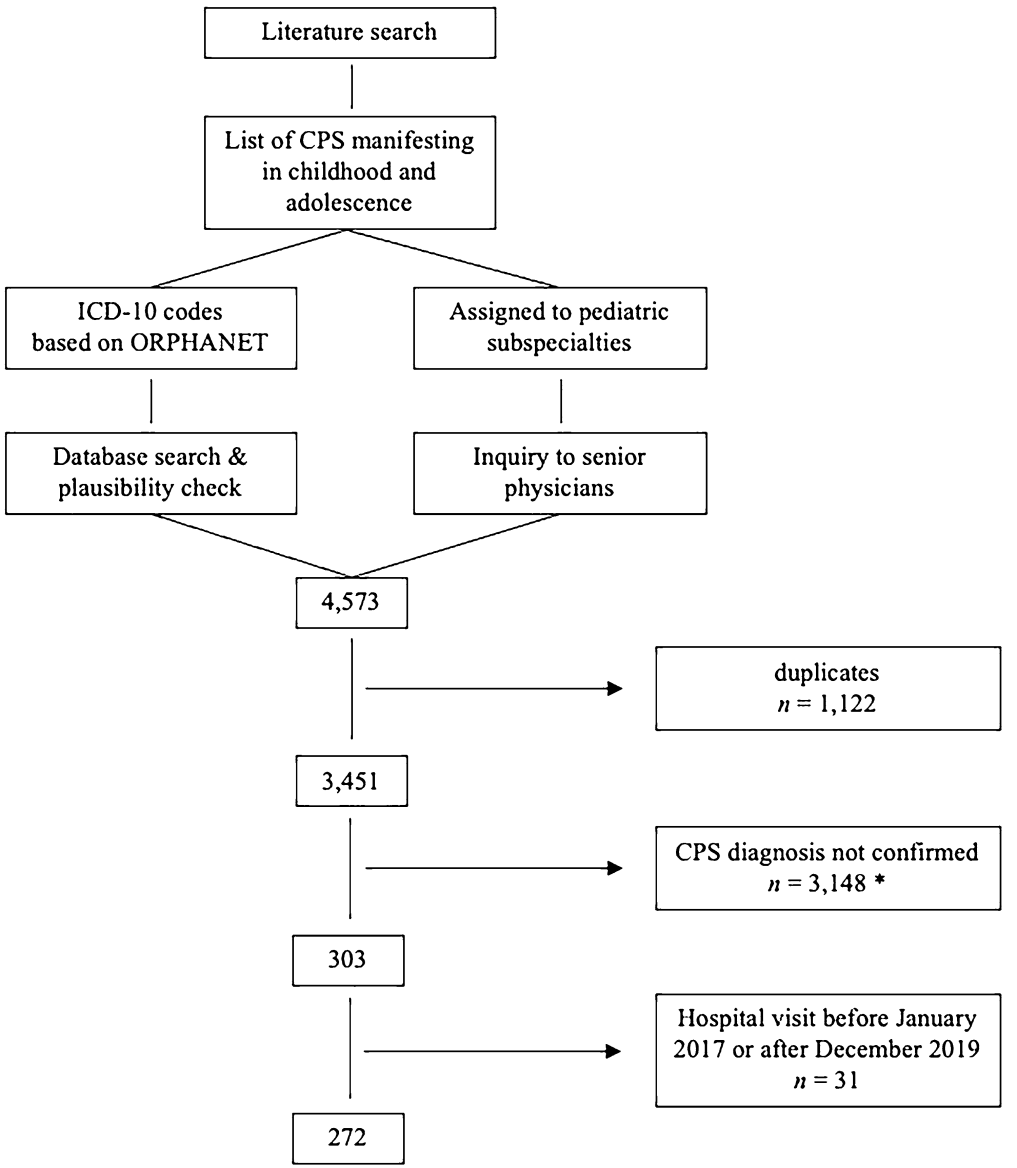
Consort diagram. *CPS diagnoses not confirmed because of imprecise coding; following manual review of medical reports

We retrieved a total of 4573 patients by ICD-10 codes; 4301 patients were ultimately excluded, 272 patients remained eligible (Fig. 1). Median age was 9.1 years (range, 0.4–27.8) on December 31, 2019. Sex ratio demonstrated a small male preponderance (144 [52.9%] males; 128 [47.1%] females). During the study period, three (1.1%) patients died of non-malignant perinatal complications of the underlying CPS. One or multiple benign and/or malignant tumors were diagnosed in 80 (29.4%) patients. Of those, all 49 patients with malignancies were seen in the department of pediatric hemato-oncology for treatment and regular follow-up, respectively. No patient died of malignancy within the study period.

**Fig. 2:**
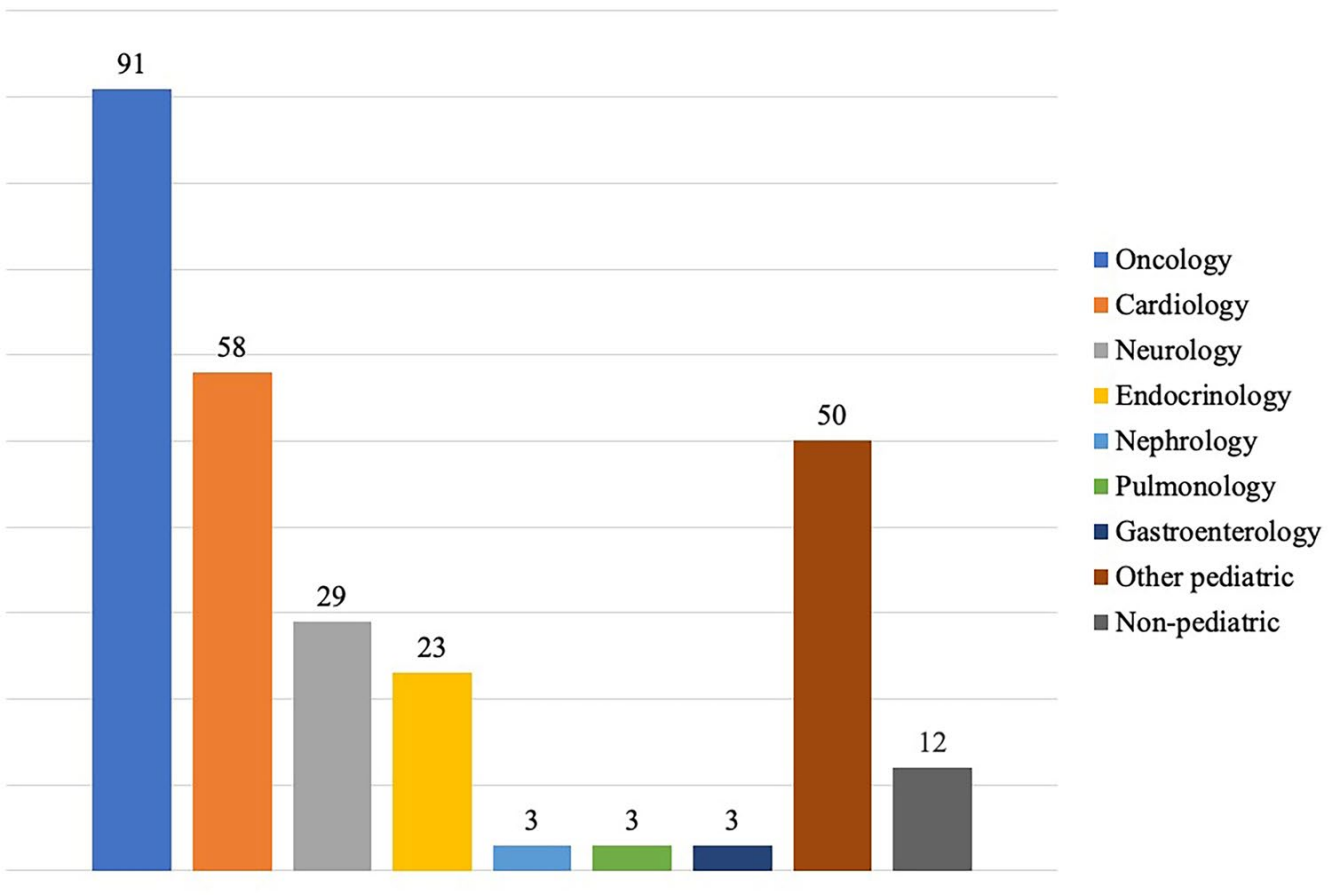
Departments of the University Medical Center caring for 272 patients diagnosed with cancer predisposition syndromes. The departments of pediatric immunology, rheumatology, radiology, emergency care, surgery, developmental pediatrics, general pediatrics, neonatology, and intensive care are summarized to “other pediatric,” the departments of dermatology, ophthalmology, and ear-nose-throat medicine to “non-pediatric”

The cohort of 272 patients presented to 20 different departments of the UMC including five non-pediatric departments. While only 18 (6.6%) patients received in-patient treatment, 254 (93.4%) received out-patient care. Of 272 patients, 194 (71.3%) were cared for in one department only; 78 (28.7%) presented to two or more departments. A total of 91 (33.5%) were primarily cared for at the department of pediatric hemato-oncology, 58 (21.3%) at the department of cardiology, and 29 (10.7%) at the department of pediatric neurology (Fig. 2). Twelve (4.4%) patients only presented to non-pediatric departments.

**Fig. 3:**
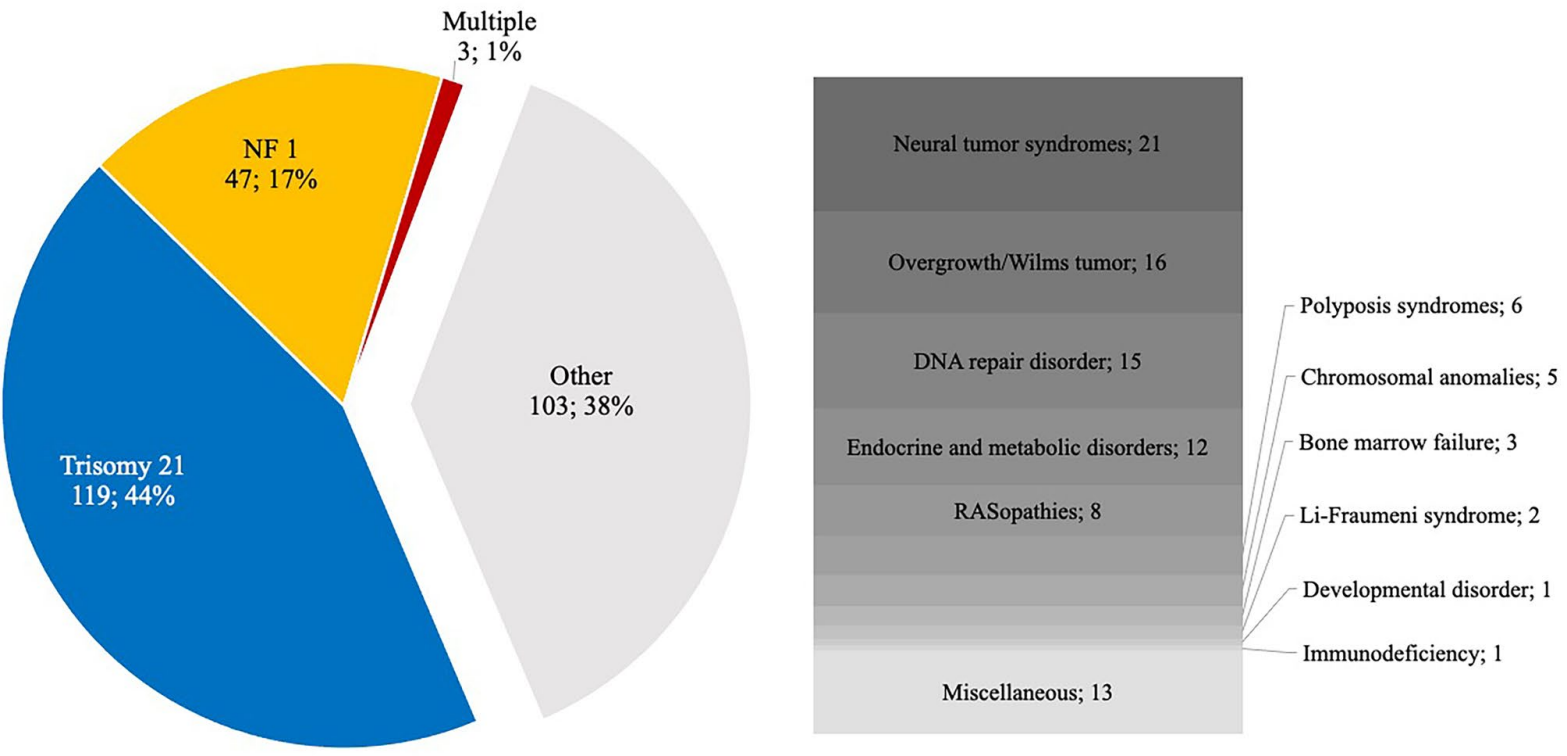
Cancer predisposition syndromes (CPS) and their relative proportion of all CPS that occurred in 272 patients treated at the University Medical Center. Rare entities were summarized to syndrome groups

A total of 41 different CPS entities were identified in 272 patients. Trisomy 21 accounted for the largest proportion (*n* = 120, 44.1%) of all cases followed by neurofibromatosis type 1 (NF1) (*n* = 48, 17.6%) and retinoblastoma predisposition (*n* = 10, 3.7%). At least three (1.1%) patients were diagnosed with multiple CPSs (patient 1, trisomy 21 and tuberous sclerosis complex; patient 2, Noonan syndrome and NF1; patient 3, 13q deletion syndrome, 17q deletion syndrome, and retinoblastoma predisposition syndrome) (Fig. 3).

Guidelines for surveillance existed for 31 (75.6%) of the 41 CPS entities. While specific surveillance was recommended in affected patients for 22 (53.6%) CPS entities, it was restricted to certain genes/variants underlying the CPS in 3 (7.3%). In 6 (14.6%) CPS entities, no explicit surveillance was recommended (Table 2).

**Table 2:** Cancer predisposition syndromes (CPS) identified in 272 patients. Indicated are the number of patients per CPS. In 3 patients, multiple CPS were diagnosed. Consensus recommendations are referenced in the column on the right. Recommendations depending on the underlying gene/pathogenic variant are marked by a sharp

Syndrome	No. of patients	Surveillance recommendations			Surveillance status					Reference
		**Recommended**	**Not recommended**	**Not stated/unclear**	**Oncological treatment or follow-up**	**Specialized CPS surveillance**	**CPS-specific** **surveillance**	**Regular consultations but no CPS-specific surveillance**	**No surveillance or status unknown**	
Trisomy 21	120	120			11	0	0	64	45	21
Neurofibromatosis type 1	48	48			19	4	7	5	13	28
Retinoblastoma predisposition	10	10			4	0	0	1	5	19
Tuberous sclerosis complex	9	9			2	0	4	0	3	-
Diamond-Blackfan anemia	8	8			0	7	0	0	1	21
Multiple endocrine neoplasia type 2A	8	8			0	3	4	0	1	27
Noonan syndrome #	7	1	1	5	1	0	0	4	2	25
Overgrowth syndrome #	7	7			0	4	0	1	2	18
Beckwith-Wiedemann syndrome	5	5			0	4	0	0	1	18
Fanconi anemia	4	4			1	2	0	0	1	21
Oculocutaneous albinism	4			4	0	0	1	1	2	
Trisomy 18	4	4			0	0	0	2	2	18
Multiple cartilaginous exostoses	3			3	0	1	0	0	2	
Peutz-Jeghers syndrome	3	3			0	0	2	0	1	16
Ataxia teleangiectasia	2	2			0	0	0	1	1	21
CLOVES syndrome	2		2		0	1	0	0	1	18
Li-Fraumeni syndrome	2	2			1	1	0	0	0	20
Lynch syndrome	2	2			2	0	0	0	0	24
Neurofibromatosis type 2	2	2			2	0	0	0	0	28
Rhabdoid tumor predisposition syndrome #	2	2			2	0	0	0	0	17
Rubinstein-Taybi syndrome	2		2		0	0	1	1	0	25
Sotos syndrome	2		2		0	0	0	0	2	25
Von-Hippel-Lindau syndrome	2	2			1	0	0	0	1	22
ATRX syndrome	1			1	0	0	0	1	0	
Carney complex	1			1	0	0	1	0	0	
Denys-Drash syndrome	1	1			0	0	1	0	0	18
DICER1 syndrome	1	1			0	1	0	0	0	23
Dyskeratosis congenita	1	1			1	0	0	0	0	21
Glykogenosis type VI	1		1		0	0	0	1	0	16
Cardiofaciocutaneous syndrome	1		1		0	0	0	1	0	25
Congenital amegacaryocytic thrombocytopenia	1			1	0	1	0	0	0	
LEOPARD syndrome	1		1		0	0	0	1	0	25
Bruton syndrome	1			1	0	0	0	0	1	
Microdeletion syndrome	1			1	1	0	0	0	0	
Polyposis coli	1	1			0	0	1	0	0	16
SAMD9L	1	1			1	0	0	0	0	21
Shwachman-Diamond syndrome	1	1			0	1	0	0	0	21
Trisomy 13	1			1	0	0	0	0	1	
WAGR syndrome	1	1			1	0	0	0	0	18
13q deletion syndrome	1			1	0	0	0	0	1	
17q deletion syndrome	1			1	0	0	0	0	1	

Patients who died during the study period (*n* = 3) and patients in oncological treatment and regular oncological follow-up, respectively, (*n* = 49) were excluded for analysis of the surveillance strategy. Of the remaining 220 (80.9%) patients, 30/220 (13.6%) were regularly assessed in a specified CPS program by the department of pediatric oncology (according to consensus recommendations), 22/220 (10.0%) regularly presented to non-oncological departments and received CPS-specific institutional surveillance not complying with consensus recommendations, 84/220 (38.2%) were regularly cared for in non-oncological departments for symptoms and coexisting conditions without receiving CPS-specific surveillance, and 84/220 (38.2%) were not regularly followed at the University Hospital Augsburg.

Patients not included in a specialized CPS program were seen a median of twice (range, 0–33) during the 3-year study period.

Surveillance recommendations were available for 209/220 (95.0%) patients. Their surveillance adhered to consensus recommendations in 54/209 (25.8%) patients, whereas it did not comply with current recommendations in 151/209 (72.2%). Of those, 149/151 (98.7%) patients did not receive surveillance or surveillance modalities were incomplete, whereas 2/151 (1.3%) patients received regular surveillance although this was not recommended. In 4/209 (1.9%) patients with Noonan syndrome, the underlying genetic variant was not documented. Thus, adherence to recent recommendations could not be determined.

Comparing adherence to details of surveillance recommendations, the surveillance program did not comply with recent recommendations in 109 of 120 (90.8%) patients with trisomy 21 and in 18 of 48 (37.5%) patients with NF1, while recommendations were followed in 7 of 8 (87.5%) patients with Diamond-Blackfan anemia. The 11/11 patients with trisomy 21 and 19/30 patients with NF1 in whom surveillance was done according to recommendations were cared for in the department of pediatric hemato-oncology.

In 9 patients, a CPS with an estimated cancer risk of 1 to 5% was identified. Of those, 2/9 (22.2%) patients (CLOVES syndrome, Rubinstein-Taybi syndrome) received surveillance, whereas 7/9 (77.8%) did not. Surveillance, however, did not comply with AACR recommendations in both patients.

## Discussion

Cancer predisposition syndromes in children and adolescents carry numerous challenges, among others a wide spectrum of rare diagnoses and coexisting conditions involving various medical specialties. Thus, an array of management and tumor surveillance issues needs to be considered. Tumor risk and surveillance recommendations may depend on the specific underlying genetic variation.

Our data mirror these challenges. We identified 272 children, adolescents, and young adults with 41 different CPS entities cared for at 20 different departments of the UMC Augsburg. In 72.2% of these patients, surveillance programs did not comply with current national or international recommendations; improvement needs to be discussed with patients and parents and details adapted to recommendations as appropriate. To the best of our knowledge, there are no other surveys reporting on children affected by CPS presenting at a distinct tertiary-care children’s hospital.

Within the past two decades, hereditary cancer predisposition management has been recognized in pediatric oncology and integrated into current care [2–5, 30, 31]. In line with this, all patients with a CPS who presented at the department of pediatric hemato-oncology of our academic center were included in a specialized CPS program according to consensus recommendations. This included patients with well-known leukemia-predisposing conditions such as Fanconi anemia, Diamond-Blackfan anemia, Shwachman-Diamond syndrome, and SAMD9L germline variants.

An increased risk for the development of tumors has been perceived by other pediatric specialists for numerous rare genetic disorders and has accordingly been integrated into their management. CPS-specific institutional surveillance initiated by non-oncological specialists was provided to 7 patients with NF1; 4 patients each with tuberous sclerosis complex and multiple endocrine neoplasia type 2A; 2 patients with Peutz-Jeghers syndrome; and one patient each with Rubinstein-Taybi syndrome, Polyposis coli, oculocutaneous albinism, Denys-Drash syndrome, and Carney complex. Noteworthy, in oculocutaneous albinism and Carney complex, surveillance recommendations are not available. Yet, surveillance in these patients may be useful and needs to be discussed on an individual basis. In fact, Carney complex predisposes to various cancers and multiple endocrine and non-endocrine tumors leading to premature death [32].

In 75.6% of the CPS entities encountered in our cohort, corresponding to 95.5% of all patients, guidelines were available, and surveillance was recommended in 243 patients, and recommended depending on the underlying variant in 9 patients. Variant-specific surveillance recommendations were available among others in multiple endocrine neoplasia type 2, von Hippel-Lindau syndrome, Noonan syndrome, and Gorlin syndrome [17, 22, 25, 27]. New insights in genotype–phenotype correlations will facilitate more genotype-specific recommendations for surveillance in the future [33].

In at least 151 patients, surveillance modalities did not comply with consensus recommendations. In most of these patients, the underlying tumor risk was only one (new) aspect of many of the primary disease but had no priority in daily care. Instead, most patients presented with serious other conditions necessitating specialized pediatric care, for example cardiac anomalies requiring cardiologic treatment in the large cohort of patients with trisomy 21 (109/151 patients, 72.2%). Patients with trisomy 21 are at a 500-fold increased risk of developing myeloid leukemia of Down syndrome [34], a nearly 20-fold increased risk of developing acute lymphoblastic leukemia [35] (corresponding to a risk of 1–2% each), and a 10% risk of transient myeloproliferative disease. The expert panel recommended regular complete blood counts in patients with trisomy 21 [21], which may easily be integrated in routine cardiologic follow-up visits and certainly does not necessitate care by a specialized cancer predisposition clinic.

Patients with NF1 present with a variable clinical phenotype and multisystem involvement commonly requiring specialist care by experienced pediatric neurologists. The highly increased risk for a wide range of malignancies has been recognized by pediatricians and requires an age-specific, regular tumor surveillance [28]. In 18/48 (37.5%) patients with NF1, however, surveillance modalities gathered from the digital records did not adhere to consensus recommendations and required improvement.

On the other hand, medical professionals were less aware of the increased risk of developing cancer in less common CPS with severe coexisting conditions such as Noonan syndrome. Due to the small number of patients, this is not provided in percentages in our study. It may, however, be of major clinical importance to every single child.

In contrast, despite not being recommended, tumor surveillance was performed in 2 patients with CLOVES syndrome and Rubinstein-Taybi syndrome, respectively. In the patient with CLOVES syndrome, this was due to the intensive wish of the patient. We were not able to assess whether in the other patient this was due to the patient’s and family’s preferences or due to lack of knowledge of consensus recommendations.

Surveillance was explicitly not recommended by the expert panel, if the cancer risk was below < 1%, or in specific conditions with a cancer risk between 1 and 5% [11]. This concerned 9 of our patients with 6 CPS entities, specifically cardiofaciocutaneous syndrome, CLOVES syndrome, Glycogenosis type VI, LEOPARD syndrome, Rubinstein-Taybi syndrome, and Sotos syndrome. The expert panel based surveillance decisions on cancer risk and the assumed benefit of early tumor detection including relatively cost-effectiveness of surveillance modalities [11]. While the cost-effectiveness of early cancer surveillance has recently been demonstrated for patients with Li-Fraumeni syndrome [36], such analyses are lacking for most CPS. In addition, tumor risk in various disorders is not well established and whether surveillance is truly useful remains speculative. Even more importantly, the physical and psychological burden of repeated examinations and cumbersome surveillance protocols placed on the patients and their families as well as the uncertainties of the yield of surveillance and side-effects need to be considered [1, 11, 37]. Van Engelken et al. recently reported on adolescents and parents for whom the benefits of surveillance outweighed perceived challenges [13]. Positive experiences were related to feelings of reassurance and taking a proactive approach. Thus, the decision for or against tumor surveillance needs to include individual patient and family preferences. Psychosocial support should be offered to families on a regular basis. Due to the study design, we were not able to analyze the psychosocial burden, supportive needs, and support in place of affected families.

Based on our review of patient records, CPS-specific surveillance was not performed in 168 patients even though national or international surveillance recommendations were available for all of these. This indicates that awareness of cancer risks and the knowledge of recommendations for the management and tumor surveillance among pediatricians is still limited. To increase awareness, we need widely published education of medical professionals as well as of patients and families on cancer risk and tumor surveillance.

In addition, the complexity of hereditary cancer predisposition necessitates a specialized multidisciplinary team of pediatric oncologists, human geneticists, and psychologists for individualized cancer risk assessment and personalized counselling of affected families. We recognize that in some countries, a specialized genetic counselor is also part of the CPS team but is not available in all countries. In recent years, a number of cancer predisposition programs and dedicated cancer predisposition clinics for children have been established. In Germany, such clinics and programs are not yet offered on a nationwide basis and need to be expanded.

Our study has several limitations. We may not be aware of CPS-specific surveillance in place by other health care providers in 84 patients cared for in non-oncological departments for symptoms and coexisting conditions and in 84 patients not regularly presenting at the UMC. In addition, patients and/or their relatives may have actively refused to participate in tumor surveillance for numerous reasons. Our approach was based on digital records. Important information, however, may not have been documented fully. The ICD-10 code system does not allow for coding rare diseases such as most CPS. Most likely, we did not identify a number of patients, especially those not receiving CPS-specific surveillance.

Nevertheless, our analysis strongly demonstrates the urgent need for evaluating current practice of tumor surveillance in children and adolescents with CPS and clearly advocates for a more systematic and consistent integration of tumor surveillance in daily practice.

The spectrum of CPS patients cared for at our tertiary care children’s hospital is manifold. In most patients, increased awareness of cancer risk is necessary and current practice needs to be adapted to published recommendations. Offering specialized CPS consultations and establishing education programs for patients, relatives, and physicians will hopefully increase adherence to surveillance recommendations.

## Data Availability

Not applicable.

## Abbreviations

AACR: American Association for Cancer Research
CPS: Cancer predisposition syndrome
NF1: Neurofibromatosis type 1
UMC: University Medical Center
